# LocusFocus: Web-based colocalization for the annotation and functional follow-up of GWAS

**DOI:** 10.1371/journal.pcbi.1008336

**Published:** 2020-10-22

**Authors:** Naim Panjwani, Fan Wang, Scott Mastromatteo, Allen Bao, Cheng Wang, Gengming He, Jiafen Gong, Johanna M. Rommens, Lei Sun, Lisa J. Strug

**Affiliations:** 1 Program in Genetics and Genome Biology, The Hospital for Sick Children, Toronto, Ontario, Canada; 2 Department of Statistical Sciences, University of Toronto, Toronto, Ontario, Canada; 3 The Centre for Applied Genomics, The Hospital for Sick Children, Toronto, Ontario, Canada; 4 Department of Molecular Genetics, University of Toronto, Toronto, Ontario, Canada; Johns Hopkins University, UNITED STATES

## Abstract

Genome-wide association studies (GWAS) have primarily identified trait-associated loci in the non-coding genome. Colocalization analyses of SNP associations from GWAS with expression quantitative trait loci (eQTL) evidence enable the generation of hypotheses about responsible mechanism, genes and tissues of origin to guide functional characterization. Here, we present a web-based colocalization browsing and testing tool named *LocusFocus* (https://locusfocus.research.sickkids.ca). LocusFocus formally tests colocalization using our established *Simple Sum* method to identify the most relevant genes and tissues for a particular GWAS locus in the presence of high linkage disequilibrium and/or allelic heterogeneity. We demonstrate the utility of LocusFocus, following up on a genome-wide significant locus from a GWAS of meconium ileus (an intestinal obstruction in cystic fibrosis). Using LocusFocus for colocalization analysis with eQTL data suggests variation in *ATP12A* gene expression in the pancreas rather than intestine is responsible for the GWAS locus. LocusFocus has no operating system dependencies and may be installed in a local web server. LocusFocus is available under the MIT license, with full documentation and source code accessible on GitHub at https://github.com/naim-panjwani/LocusFocus.

This is a *PLOS Computational Biology* Software paper.

## Introduction

The majority of disease-associated variants identified by genome-wide association studies (GWAS) lie in non-protein-coding regions of the genome [1]. Non-coding GWAS variants may tag *cis*-regulatory elements that impact gene expression [2], offering hypotheses on underlying mechanisms that influence a disease phenotype. Integrating GWAS summary statistics with functional datasets such as expression quantitative trait locus (eQTL) data is an integral next step to guide functional studies.

Several summary statistic-based colocalization methods are in use, such as coloc [3], eCAVIAR [4], RTC [5], Enloc [6], COLOC2 [7], and SMR-multi [8]. Common challenges for these tools include 1) the impact of linkage disequilibrium (LD), 2) allelic heterogeneity, and 3) the absence of causal variants in the dataset (untyped or not called) [9].

The *Simple Sum (SS*) [10] is a frequentist colocalization method that is more powerful for colocalization than existing methods, and in particular in regions of high LD and allelic heterogeneity. Benchmarking of the *SS* performance relative to other methods is extensively documented in [10]. When integrating an eQTL dataset, the *SS* method determines whether a GWAS signal is driven by expression variation and prioritizes the most probable responsible gene(s) and tissue(s) at the locus. In our previous work on a GWAS of meconium ileus (MI) [10], an intestinal obstruction phenotype in individuals with cystic fibrosis (CF), we showed how the *SS* guided the identification of the likely responsible gene(s) for each genome-wide significant locus and pointed to the pancreas as a common contributor in the pathophysiology of MI, a CF phenotype that manifests in the intestine. For example, the genome-wide significant signal detected around the *ATP12A* gene clearly showed colocalization with GTEx eQTLs of *ATP12A* [11] in the pancreas, and only the *SS* colocalization method highlighted the colocalization, while no support for other digestive system tissues was evident. Here we make visualization and testing of colocalization via the *SS* method accessible in a web application named LocusFocus (https://locusfocus.research.sickkids.ca).

LocusFocus allows the user to upload GWAS summary statistics and any other secondary SNP-level summary statistic dataset (e.g. eQTL, mQTL or other GWAS associations) to test colocalization at a particular locus (S1 Fig). In the example shown, the primary dataset is a GWAS locus for MI and the secondary datasets are eQTL p-values from GTEx or those from our own study of primary nasal epithelia (HNE) from individuals with CF. We have made eQTL summary statistics from GTEx (v7 and v8) available for selection within our web server to easily test colocalization with GTEx tissues and genes using the *SS* method.

## Design and implementation

**Fig 1:**
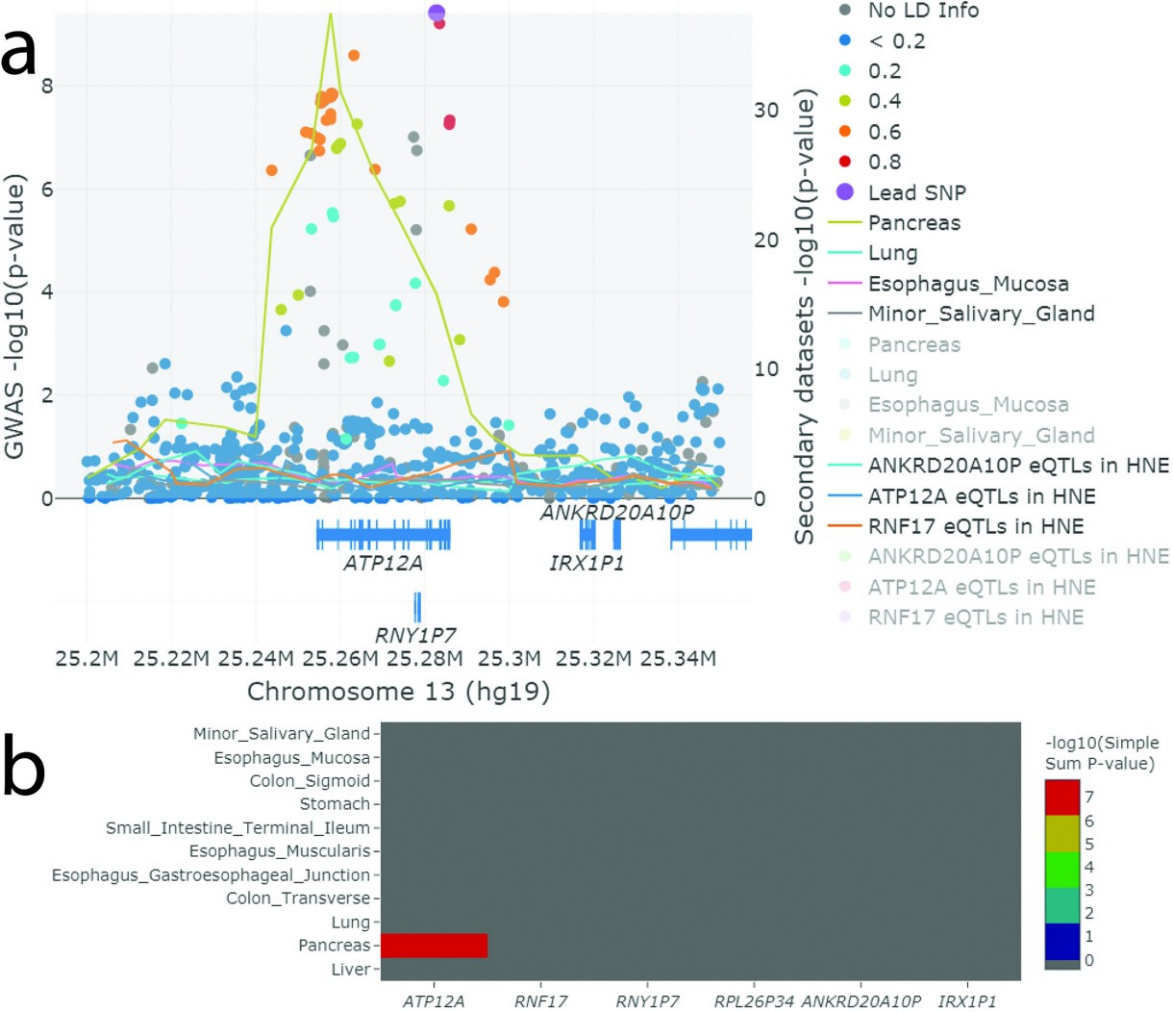
Sample interactive plot output from the LocusFocus web application. GWAS summary statistics of MI in individuals with CF for chr13q12.12 and eQTLs from HNEs from individuals with CF were uploaded, and digestive tissues and lung from GTEx were selected for colocalization analysis (interactive plots available at bit.ly/LocusFocus-*ATP12A*-Example). A more detailed explanation of all components of the figure is provided in S2A Fig**)** Filled circles represent GWAS -log_10_(p-values) (left y-axis) for MI. Lines (right y-axis) serve as a visual guide of the secondary datasets and trace the lowest p-value per 22.5bp window. Gene track is from GENCODE v19, with transcripts collapsed into single genes. The gray shaded region shows the region used for the *SS* calculation, 0.1 Mbp on each side of the selected lead SNP is the default. We used the full region for the *SS* calculations. Users may click the tissue panel list in the legend to show or hide information. The eQTL scatterplots, from which the line traces are derived from, are hidden by default but may be overlaid by clicking on the grayed-out text in the legend. All tissues were tested (S1 Table and S2 Fig, or view interactively at bit.ly/LocusFocus-*ATP12A*-Full-Example). Other features of the plot include the ability to zoom in, tooltips for each data point, save image options in png or svg vector format, selection and fading tools, and resetting, rescaling or shifting of axes. **b**) The heatmap shown summarizes the *SS* colocalization tests for all the genes in the user-defined region and across all the selected tissues. Gray squares indicate either no eQTL data (typically due to little or no expression), or the gene-tissue pair does not have significant eQTL signal after Bonferroni correction (see S1 Table for exact reason). **C**olocalization for eQTLs in HNEs are summarized as an interactive table online and were either not significant or were not expressed for all six genes (S2 Fig).

LocusFocus is a web application which uses Python’s Flask as the underlying app engine for *SS* colocalization analysis and subsequent visualization. GTEx v7 and v8 [11] eQTL summary statistics for all 49 tissues are indexed and stored in a MongoDB database to enable efficient querying. The plots employ Plotly.js (https://plot.ly/javascript/) to enable interactivity with the data. S1 Fig displays the LocusFocus web interface and required input fields. After submission of the user’s GWAS summary data, colocalization analysis [using SS [10] and COLOC2 [7]] is first performed and colocalization and heatmap plots are then generated for the visualization of GWAS, eQTL, and optional secondary datasets uploaded by the user (Fig 1). Calculation of the LD matrix based on the 1000 Genomes (phase 1, version 3) [12] is performed on demand for the user-specified region and SNPs using PLINK v1.90b6.9 [13]. Alternatively, the user may input their own population-specific PLINK-generated LD matrix. Users’ integrated data are stored in sessions with unique identifiers for easy sharing of the session data and plots (stored at least 7 days). The gene track shown below the plots (Fig 1A) is from GENCODE v19 when using hg19 coordinates and v26 when using hg38 coordinates [14], customized to collapse transcript isoforms into single gene models; the gene coordinates were downloaded from GTEx’s web portal.

## Results

### Colocalization analysis with LocusFocus

GWAS summary statistics for MI at the chr13q12.12 (chr13:25.20–25.35Mbp; hg19) locus, near *ATP12A*, were uploaded into LocusFocus. GWAS summary statistics, LD matrix and eQTL data are integrated with outputs of interactive colocalization, heatmap plots (Fig 1), and interactive summary tables (S1 Table). Results support strong colocalization for *ATP12A* in the pancreas as reported [10]. Interestingly, *ATP12A* has been proposed as a modifier of lung disease severity via its role in pH regulation [15]. Our GWAS on lung disease severity in CF, however, revealed no association at this locus [16]. In the event that the CF lung GWAS was confounded, we tested colocalization at the CF MI GWAS locus with eQTLs in lung from GTEx and eQTLs from RNAseq of HNEs harvested from individuals with CF as described in [17] and imputed using a hybrid reference sequence using the 1000 Genomes and 101 individuals with CF as described in [18]. HNE eQTLs for *ATP12A* did not colocalize with the MI GWAS locus (Fig 1). Of note, the current analysis is limited by the tissue sampling, which is confined by source and cell types present. Colocalization applications clearly benefit from the best datasets available.

### Availability and future directions

The datasets used, and detailed examples, are available on the LocusFocus GitHub repository (https://github.com/naim-panjwani/LocusFocus; under data/sample_datasets folder). The datasets and file names used are as follows:

- MI GWAS around *ATP12A*: MI_GWAS_2019_13_25180-25400kbp.tsv (subset from the main MI GWAS study [10])
- Secondary HNE eQTL data: atp12a_HNE_eqtl.html
- GTEx eQTL data: available on the GTEx portal, and indexed as a NoSQL database within our web server to enable easy querying from our tool
- The session generated has been archived and is available at bit.ly/LocusFocus-*ATP12A*-Example and bit.ly/LocusFocus-ATP12A-Full-Example

These datasets use the hg19 coordinate system. Although LocusFocus allows the user to choose hg38 and hg19 as the input coordinates, co-localization analysis does not directly depend on the coordinate system. The user is required to input a primary dataset of summary statistics, and one or more secondary datasets to compare with, making sure the data sets use the same coordinate system.

More examples on the usage of LocusFocus are available in the online documentation (https://locusfocus.readthedocs.io/en/latest/examples.html) as are a list of planned improvements (https://locusfocus.readthedocs.io/en/latest/future.html).

Important future updates will enable uploading of compressed files, a queue system for job submission and later retrieval, and implementation of the SMR-multi [8] more colocalization methods.

## Supporting information

S1 Fig LocusFocus Web Application Input Form. A web-based input form is presented to the user to upload datasets for colocalization analysis at https://locusfocus.research.sickkids.ca. **a)** The Session ID button allows the user to retrieve previous colocalization analyses. These sessions are currently stored for at least 7 days. Easy navigation to documentation and example output is provided. **b)** Selection of the hg19 or hg38 coordinate systems changes the form to enable selection of hg19- or hg38-aligned 1000 Genomes and either GTEx v7 (hg19) or GTEx v8 (hg38) data. **c)** An upload button is provided for up to 3 files not exceeding 100 MB in total (at least the first file is required). File extensions dictate the type of file uploaded: 1).txt and.tsv files are assumed to be summary statistics for the primary dataset to test colocalization with and is required; this is usually a GWAS dataset. Optionally, one may upload 2) the LD matrix output from PLINK (—r2 square;.ld file extension) and or 3) a multi-sample dataset formatted in HTML format with the secondary summary statistics at the same locus as the primary dataset to test colocalization with. **d)** Column names for the primary dataset may be changed here. A minimum of two columns, in any order in the file, are required when the “Use marker ID column to infer variant position and alleles” checkbox is checked (the marker column name with rsid or chrom_pos_ref_alt_b37/b38, and a p-value column). When only the variant ID column is provided, they are mapped internally using a tabix-indexed dbSNP151 file. For better variant matching, the user may provide the chromosome, position, reference and alternate columns. COLOC2 [7] requires more variables, and checking the option to “Add required inputs for COLOC2” will request for the following additional column names: beta, standard error, total number of samples, minor allele frequency and study type. In the case of a case-control study type, the number of cases is required as input as well. The coordinates to view plot results are also required (limited to 2 Mbp regions). The lead SNP with the lowest p-value is chosen as default but the user may input an alternate lead SNP. If the 1000 Genomes is used for the LD matrix, and the lead SNP is not found in the 1000 Genomes, we iterate in ascending p-value order until a SNP in both 1000 Genomes and input dataset is found for pairwise LD. **e)** The Simple Sum (*SS)* tests colocalization across a default region of 0.1 Mbp on either side of the lead SNP, but the user may input a customized region up to 2 Mbp (the evaluated area will appear in gray shading in the first plot output). **f)** Can be ignored if a user.ld file was provided in B, otherwise, the 1000 Genomes population [12] that most closely resembles the input dataset may be selected. **g)** Secondary datasets from any-any subgroup or all 48 tissues from GTEx (v7) [11] are available for selection within the webserver. Genes that fall within the region provided in **d** are available for selection and colocalization testing. All genes are made available for browsing in the colocalization plot in the output page via a dropdown. Colocalization is tested for each of the tissues and genes selected.
(TIF)

S2 Fig Sample interactive plot output from the LocusFocus web application. Same as Fig 1, but including all GTEx tissues and HNEs from individuals with CF. GWAS summary statistics of MI and lung disease severity in patients with CF for chr13q12.12 and eQTLs from HNEs from individuals with CF were uploaded, and all tissues from GTEx [11] were selected for colocalization analysis. The plots are traced using plotly in JavaScript (https://plot.ly/javascript/) after merging of the input data. The interactive plot is available at bit.ly/LocusFocus-ATP12A-Full-Example. **a)** Filled circles represent GWAS data (with corresponding y-axis on the left) for MI. The LD information presented is similar to LocusZoom [19] (lead SNP in purple, high LD SNPs with r^2^ ≥ 0.8 in red with the lead SNP, orange for 0.8 < r^2^ ≤ 0.6, green for 0.6 < r^2^ ≤ 0.4, light blue for 0.4 < r^2^ ≤ 0.2 and dark blue for r^2^ < 0.2; markers with no LD information are shown in gray). LD information was computed from the European 1000 Genomes subset (phase 1, version 3) [12]. The web server computes the LD matrix with the 1000 Genomes on demand using PLINK v1.90b6.9 [13]. Lines shown on the plot represent a summary of GTEx (v7) and primary human nasal epithelial cells’ (HNEs) eQTL p-values for *ATP12A*, a gene proposed as a modifier for CF [15, 20, 21] (with corresponding y-axis on the right), with each line representing a tissue (eQTLs for other genes within the region can be selected and the plot re-drawn within the same session). Line traces for some tissues do not appear due to no eQTL data for *ATP12A* for that tissue (likely due to little or no expression). The lines trace the lowest p-value per window, and the windows are defined as (region size/1,000,000) × 150, where region size is the size of the region input in base pairs (up to 2 Mbp regions are allowed). A different window size can be specified and lines redrawn on the web tool. We find these parameters best illustrate the overall pattern of eQTL association for a particular window size up to 2 Mbp. Gene track information is from GENCODE v19 (hg19 coordinates), with transcripts collapsed into single genes (as described by GTEx). The gray shaded region shows the region used for the *SS* calculation, 0.1 Mbp on each side of the selected lead SNP (by default unless set differently by user). We used the full region (chr13:25,200,000–25,350,000) for the *SS* calculations. Users may click the tissue panel list in the legend to show or hide particular groups of information. The eQTL scatterplots for each tissue, from which the line traces are derived from, are hidden by default (grayed out in the legend) but may be displayed by clicking on the desired tissue in the legend (tissues listed in faint gray; not all tissues are shown in the colocalization figure above due to space; for a complete table of colocalization results, refer to S1 Table, or view interactively at bit.ly/LocusFocus-ATP12A-Full-Example). Other features of the plot include the ability to zoom in, tooltips for each data point, save image options in png or svg vector format, selection and fading tools, and resetting, rescaling or shifting of axes. **b)** The heatmap shown summarizes the *SS* colocalization tests for all the genes in the user-defined region and across all the selected GTEx tissues. Gray squares with negative p-values for colocalization indicate either no eQTL data (typically due to little or no expression), or the gene-tissue pair does not have significant eQTL signal after Bonferroni correction, or insufficient SNPs are provided for an accurate calculation of the Simple Sum p-value (the exact reason can be viewed in the web session as an interactive table output, or in S1 Table). **c)** Custom eQTL data analyzed in HNE tissue from patients with CF are output as an interactive table, and did not pass the Bonferroni-corrected first stage testing among all the secondary datasets chosen.
(TIF)

S1 Table Simple Sum (*SS*) colocalization tests for the Genome-wide Association Study (GWAS) of Meconium Ileus (MI) in individuals with Cystic Fibrosis (CF) at the *ATP12A* (chr13q12.12) locus with all GTEx (v7) tissues, and primary human nasal epithelia (HNE) from individuals with CF. Cell values are -log_10_(*SS* p-values). Values below were extracted from the LocusFocus web application (bit.ly/LocusFocus-ATP12A-Full-Example). Strength of colocalization is coloured from green (low -log_10_P) to red (high -log_10_P). Results support a strong colocalization of *ATP12A* eQTLs in the pancreas with the GWAS of MI. Gene/tissue cells described as “No eQTL data” (output as -1 by LocusFocus) have no eQTLs calculated by GTEx, likely due little or no expression; “No significant eQTLs” (output as -2 by LocusFocus) describes the scenario where eQTL data is available, but the overall eQTL p-values in relation to other eQTLs does not pass a Bonferroni-corrected threshold prior to *SS* colocalization testing; a third scenario (which does not occur in this table) for a missing *SS* p-value is “*SS* test failed” (output as -3 by LocusFocus), which is often due to an insufficient number of SNPs for a confident assessment of the *SS* colocalization test.
(DOCX)

S1 Data Figures and results in this manuscript may be re-created using the sample datasets provided with this manuscript. The tab-separated file includes the GWAS summary statistics for meconium ileus [10], and serves as the primary dataset. The html file includes eQTL summary statistics in human nasal epithelia for three genes in the chr13q12.12 (chr13:25.20–25.35Mbp; hg19) associated locus, and serves as a custom secondary dataset that may be uploaded to LocusFocus for colocalization analysis (note that while there are six genes at the locus, three of these genes did not have detectable expression and hence no eQTL results).
(RAR)
