# Supplementary figures and images for: LocusFocus: Web-based colocalization for the annotation and functional follow-up of GWAS
Source: PLoS Comput Biol. 2020 Oct 22;16(10):e1008336. doi: 10.1371/journal.pcbi.1008336 (PMC7608978; doi:10.1371/journal.pcbi.1008336)

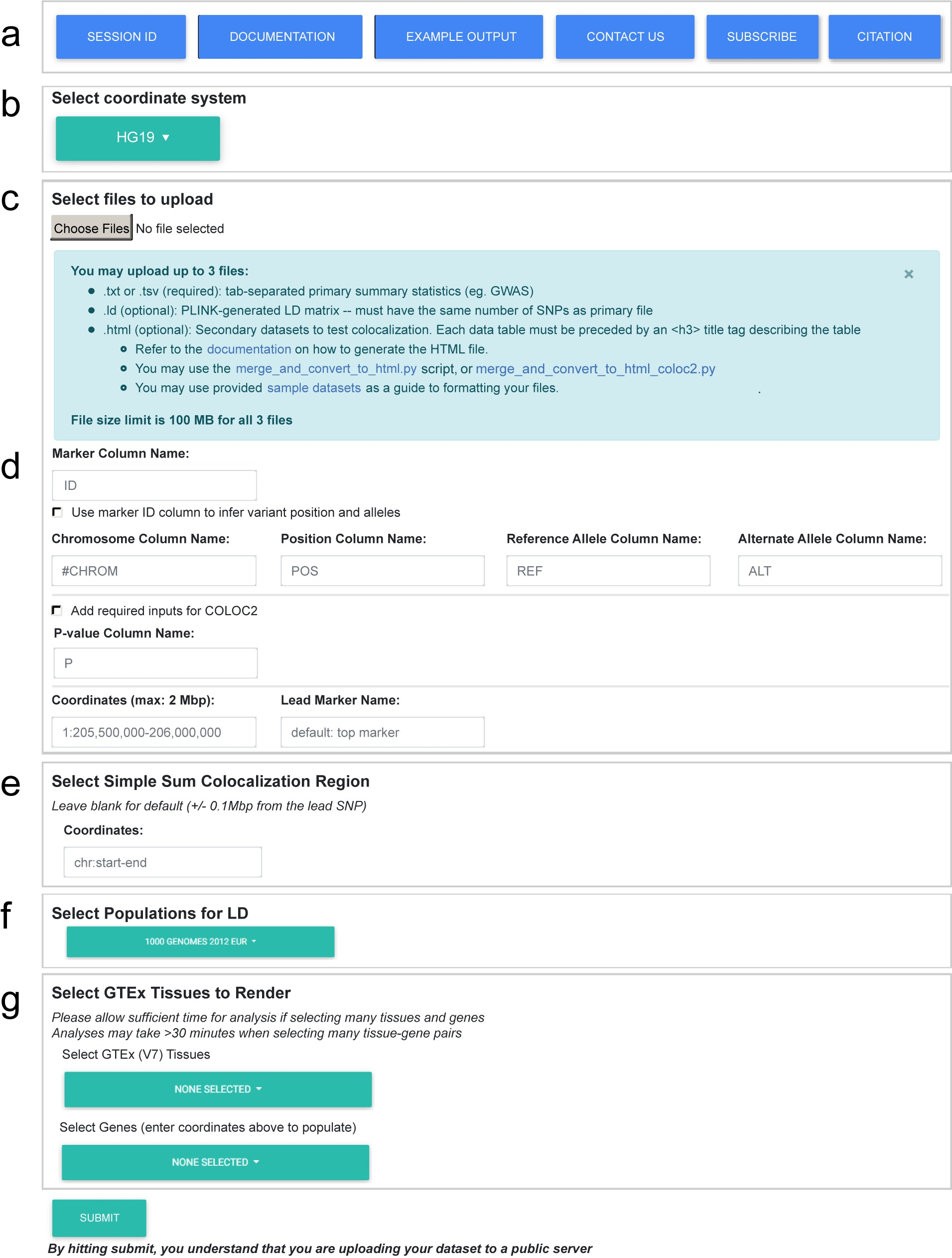

Supplement: S1 Fig — A web-based input form is presented to the user to upload datasets for colocalization analysis at https://locusfocus.research.sickkids.ca. a) The Session ID button allows the user to retrieve previous colocalization analyses. These sessions are currently stored for at least 7 days. Easy navigation to documentation and example output is provided. b) Selection of the hg19 or hg38 coordinate systems changes the form to enable selection of hg19- or hg38-aligned 1000 Genomes and either GTEx v7 (hg19) or GTEx v8 (hg38) data. c) An upload button is provided for up to 3 files not exceeding 100 MB in total (at least the first file is required). File extensions dictate the type of file uploaded: 1).txt and.tsv files are assumed to be summary statistics for the primary dataset to test colocalization with and is required; this is usually a GWAS dataset. Optionally, one may upload 2) the LD matrix output from PLINK (—r2 square;.ld file extension) and or 3) a multi-sample dataset formatted in HTML format with the secondary summary statistics at the same locus as the primary dataset to test colocalization with. d) Column names for the primary dataset may be changed here. A minimum of two columns, in any order in the file, are required when the “Use marker ID column to infer variant position and alleles” checkbox is checked (the marker column name with rsid or chrom_pos_ref_alt_b37/b38, and a p-value column). When only the variant ID column is provided, they are mapped internally using a tabix-indexed dbSNP151 file. For better variant matching, the user may provide the chromosome, position, reference and alternate columns. COLOC2 [7] requires more variables, and checking the option to “Add required inputs for COLOC2” will request for the following additional column names: beta, standard error, total number of samples, minor allele frequency and study type. In the case of a case-control study type, the number of cases is required as input as well. The coordinates to view plot result [file pcbi.1008336.s001.tif]

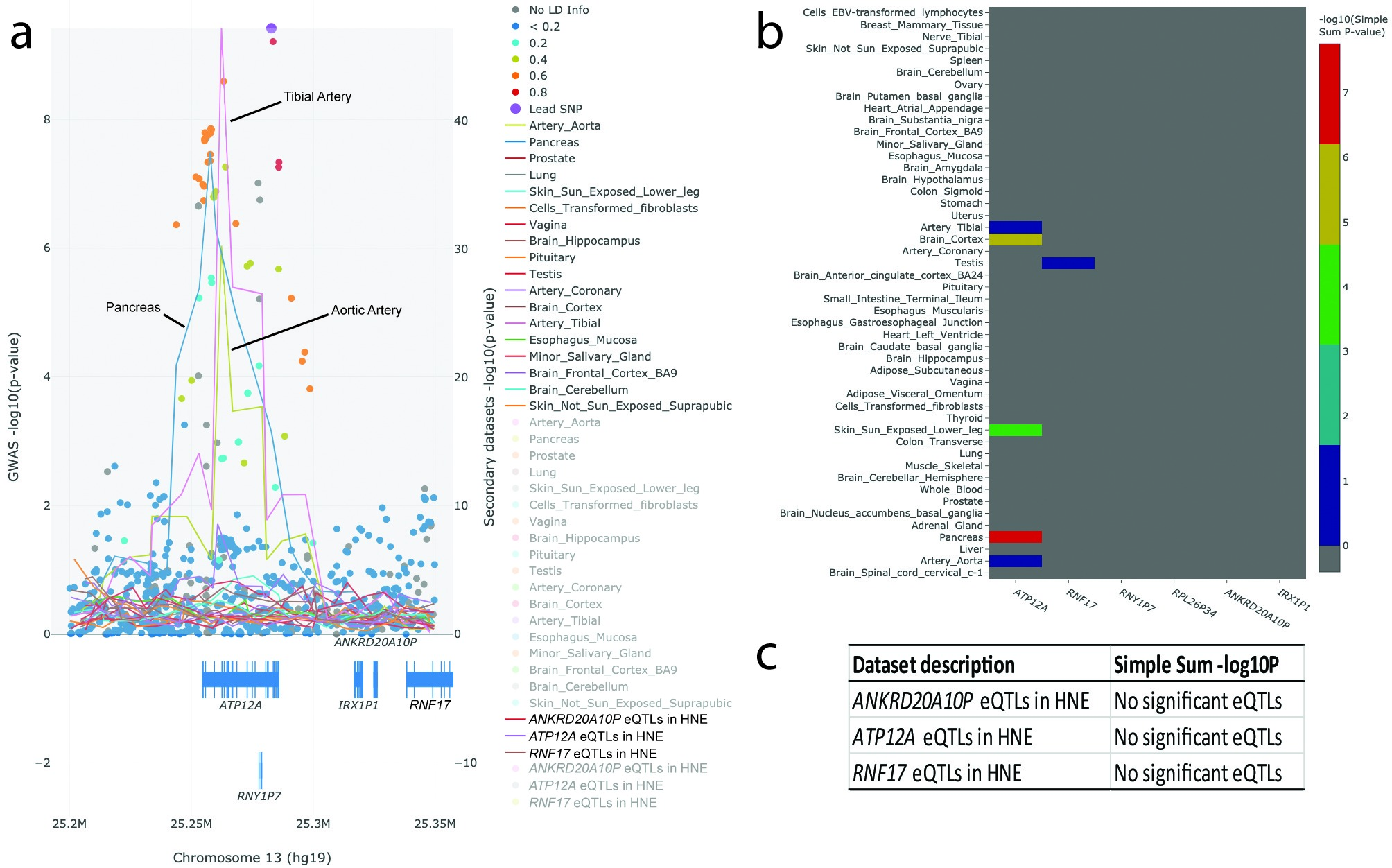

Supplement: S2 Fig — Same as Fig 1, but including all GTEx tissues and HNEs from individuals with CF. GWAS summary statistics of MI and lung disease severity in patients with CF for chr13q12.12 and eQTLs from HNEs from individuals with CF were uploaded, and all tissues from GTEx [11] were selected for colocalization analysis. The plots are traced using plotly in JavaScript (https://plot.ly/javascript/) after merging of the input data. The interactive plot is available at bit.ly/LocusFocus-ATP12A-Full-Example. a) Filled circles represent GWAS data (with corresponding y-axis on the left) for MI. The LD information presented is similar to LocusZoom [19] (lead SNP in purple, high LD SNPs with r2 ≥ 0.8 in red with the lead SNP, orange for 0.8 < r2 ≤ 0.6, green for 0.6 < r2 ≤ 0.4, light blue for 0.4 < r2 ≤ 0.2 and dark blue for r2 < 0.2; markers with no LD information are shown in gray). LD information was computed from the European 1000 Genomes subset (phase 1, version 3) [12]. The web server computes the LD matrix with the 1000 Genomes on demand using PLINK v1.90b6.9 [13]. Lines shown on the plot represent a summary of GTEx (v7) and primary human nasal epithelial cells’ (HNEs) eQTL p-values for ATP12A, a gene proposed as a modifier for CF [15, 20, 21] (with corresponding y-axis on the right), with each line representing a tissue (eQTLs for other genes within the region can be selected and the plot re-drawn within the same session). Line traces for some tissues do not appear due to no eQTL data for ATP12A for that tissue (likely due to little or no expression). The lines trace the lowest p-value per window, and the windows are defined as (region size/1,000,000) × 150, where region size is the size of the region input in base pairs (up to 2 Mbp regions are allowed). A different window size can be specified and lines redrawn on the web tool. We find these parameters best illustrate the overall pattern of eQTL association for a particular window size up to 2 Mbp. Gene track information is from [file pcbi.1008336.s002.tif]
